# The predictive value of 24-hour urinary calcium for kidney stone risk in primary hyperparathyroidism: insight from a retrospective study of parathyroid adenoma cases

**DOI:** 10.3389/fendo.2025.1671905

**Published:** 2025-11-25

**Authors:** Arti Bhan, Rebecca Simon, Aseel Yaseen, Bernard Cook, Shijing Qiu, Sudhaker D. Rao

**Affiliations:** 1 Division of Endocrinology, Diabetes, and Bone & Mineral Disorders, Detroit, MI, United States; 2 Bone and Mineral Research Laboratory, Henry Ford Health, Detroit, MI, United States; 3 Department Pathology, Henry Ford Health, Detroit, MI, United States; 4 Michigan State University College of Human Medicine, Lansing, MI, United States

**Keywords:** hypercalcemic primary hyperparathyroidism, kidney stones, 24-hour urine calcium excretion, hypercalciuria, demographic factors

## Abstract

**Background:**

Primary hyperparathyroidism (PHPT) is associated with an increased risk of kidney stones. However, the clinical utility of 24-hour urinary calcium (24h-UCa) as a predictor of nephrolithiasis, and its role in surgical decision-making, remains uncertain due to inconsistent findings.

**Objective:**

To evaluate whether 24h-UCa is a reliable disease-specific predictor of kidney stone risk in patients with PHPT and to identify demographic and biochemical determinants of urinary calcium excretion.

**Methods:**

This retrospective study included 306 PHPT patients who underwent curative parathyroidectomy for confirmed adenoma. Demographics, kidney stone history, and biochemical data were collected. T-tests, chi-square tests, multivariate logistic regression, and multiple regression were used to analyze associations of 24h-UCa with kidney stones, demographic, and biochemical indices.

**Results:**

Kidney stones were present in 22% of patients. No significant difference in 24h-UCa was observed between stone-formers and non-stone-formers, even after adjustment for age, gender, and race. In contrast, 24h-UCa was significantly higher in men and white patients, with hypercalciuria more prevalent among white individuals. Serum calcium and eGFR were also significantly positively associated with 24h-UCa.

**Conclusion:**

Our results suggest that 24h-UCa is not an independent predictor of kidney stone risk in PHPT and is largely influenced by demographic and biochemical factors. Accordingly, routine 24h-UCa measurement for evaluating patients with sporadic PHPT or for guiding parathyroidectomy decisions is not recommended. This study is limited by its retrospective design, reliance on a single urine collection, and lack of detailed dietary or genetic data, which may have introduced variability and reduced power to detect weak associations.

## 1 Introduction

Hypercalcemic primary hyperparathyroidism (PHPT) is the third most common endocrine disorder and is characterized by elevated serum calcium levels with high or inappropriately normal serum parathyroid hormone (PTH) levels (1–3). Hypercalcemia, regardless of its cause, necessarily increases the glomerular filtered load of calcium and consequent rise in urine calcium. Elevated PTH, as occurs in PHPT, increases renal tubular reabsorption of calcium, partially negating the rise in urine calcium due to higher filtered load. When the filtered load exceeds the tubular maximum for calcium reabsorption, urine calcium will rise, usually when serum calcium is >12 mg/dl. In addition, the amount of calcium excreted in the urine varies widely depending on calcium and sodium intakes, serum calcium level, gender and ethnicity. Thus, the 24-hour urinary calcium (24h-UCa) is not a reliable indicator of PHPT severity as is commonly assumed.

One of the known complications of PHPT is kidney stone formation (nephrolithiasis), and to a lesser extent nephrocalcinosis, which can significantly impact patient health and quality of life (4–6). Clinical guidelines—including those from the 5th International Workshop on PHPT, the Endocrine Society, and the American Association of Endocrine Surgeons—recommend thorough biochemical evaluations to assess the risk of kidney stones in PHPT (7, 8). Given the higher prevalence of nephrolithiasis in patients with PHPT, it is crucial to identify reliable diagnostic measures in evaluating and mitigating this risk. The most commonly used diagnostic tool is the measurement of 24h-UCa excretion to evaluate calcium metabolism (9, 10).

Nevertheless, the relationship between 24h-UCa levels and kidney stone risk in PHPT still remains unclear. Some studies have shown a correlation between elevated 24h-UCa levels and an increased risk of nephrolithiasis (11, 12), while others have not consistently found this association (13, 14). Additionally, the variability in methods used to measure 24 h-UCa and define hypercalciuria complicated the interpretation of these findings. Given the inconsistent evidence regarding the predictive capacity of 24-hour-UCa in PHPT (14–16), we attempted to determine whether 24-hour-UCa serves as an independent predictor of kidney stone formation in patients with PHPT undergoing parathyroidectomy. We hypothesized that 24h-UCa would not exhibit a significant association with kidney stone status after controlling for demographic and biochemical variables.

## 2 Materials and methods

### 2.1 Patient characteristics

PHPT in this study was defined by unambiguous hypercalcemia with either elevated or non-suppressed serum PTH levels. We included only those patients with surgically confirmed PHPT due to parathyroid adenoma(s) with no evidence of recurrence or persistence of PHPT after at least one year of follow-up evaluation. Three hundred and six adult patients with PHPT, as defined, underwent parathyroidectomy at Henry Ford Health between January 1, 2005, and December 31, 2015. We excluded patients 1) with normocalcemic hyperparathyroidism (15), 2) taking medications that are known to affect calcium homeostasis, 3) with kidney stones without PHPT; and 4) other than black and white people, due an insufficient number of other ethnic groups, 5) with familial PHPT syndromes, 6) with secondary or tertiary hyperparathyroidism (HPT), and 7) with chronic kidney disease, as assessed by serum creatinine level >1.5 mg/dl). Demographic (age, race, and gender), surgical (parathyroid adenoma), medical history (presence or absence of kidney stone history), and relevant biochemical data were extracted from the participant’s electronic health records.

### 2.2 Biochemical measurements and kidney stone assessment

Serum calcium (Ca), creatinine (Cr), and 24 h-UCa, Cr, and Sodium (Na) were measured in the Hospital laboratory by standard methods. The estimated glomerular filtration rate (eGFR) was calculated using the MDRD formula. Serum 25-hydroxyvitamin D (25(OH)D) was measured by competitive immunoassay using sheep monoclonal anti-25(OH)D antibody on Beckman Coulter UniCel Dxl platform. Serum PTH was measured by chemiluminescent assay on Roche-Cobas e801 platform. The coefficient variation ranged from 1.9-3.4% for 25(OH)D and 3.0-3.4% for PTH depending upon the concentrations of the respective hormone levels. The reference range for 24h-UCa is 50–300 mg/day for men and 50–250 mg/day for women. All the measurements closest to the parathyroidectomy (PTX) date were selected for each patient.

The kidney stone information was collected from the patient’s history during the period from recruitment into the study through the end of surgery. All data were gathered within 2 years following the detection of PHPT. Stones identified before the detection of PHPT were not included in this study. Kidney stones were diagnosed using ultrasound or CT scan.

### 2.3 Statistical analysis

Numerical data are expressed as mean and standard deviation (SD) and compared across kidney stone presence, gender, and race using Student’s t-tests, with the Mann–Whitney test applied for non-normally distributed variables. Categorical variables were analyzed using chi-square tests. Multivariate logistic regression analysis was performed to assess the relationship between 24h-UCa levels and kidney stone presence, adjusting for gender, race, and age. Multiple regression analysis was utilized to predict the demographic and serum biochemical factors that influence 24 h-UCa levels. We applied the Benjamini–Hochberg procedure to control the false discovery rate at 5% (16, 17).

## 3 Results

The cohort of 306 PHPT patients exclusively included black and white individuals with benign parathyroid adenoma. Among these, 66 (22%) were women, and 240 (78%) were white people. In addition, 66 (22%) of PHPT patients presented with or had a history of kidney stones. **Table 1** shows that patients with kidney stones were significantly older (67.3 years) than those without stones (64.0 years). The proportion of men in the group with kidney stones (32%) was significantly higher than that in the group without kidney stones (19%)(p < 0.05), suggesting that male gender might be a risk factor for kidney stone formation. Although the proportion of white people in the group with kidney stones (76%) was higher than that in the group without kidney stones (63%), the difference did not quite reach significance (p = 0.06). None of the serum biochemical variables, including Calcium, Creatinine, eGFR, PTH, and 25(OH)D, showed statistically significant differences between stone and no-stone groups (p > 0.05)(**Table 1**).

**Table 1:** Differences in demographic and serum biochemical variables between the PHPT patients with and without kidney stones.

Variable	Stone	No Stone	p
	n = 66	n = 240	
Age (years)	67.3 (10.5)	64.0 (12.3)	0.024
Gender			
Men	21 (32%)	45 (19%)	0.023
Women	45 (68%)	195 (81%)	
Race			
Black	16 (24%)	88 (37%)	0.060
White	50 (76%)	152 (63%)	
Serum Ca (mg/dL)	10.9 (0.590)	11.0 (0.587)	0.431
Serum P (mg/dL)	2.95 (0.628)	2.88 (0.555)	0.524
Serum Cr (mg/dL)	0.870 (0.230)	0.900 (0.250)	0.439
eGFR (mL/min/1.73 m^3^)	81.3 (19.9)	82.1 (21.3)	0.648
Serum PTH (pg/mL)	110 (56.0)	115 (68.5)	0.649
Serum 25(OH)D (ng/mL)	29.6 (13.8)	25.9 (10.8)	0.056

Numerical data expressed as mean (SD).

Contrary to the pervasive general assumption and expectation, there was no significant difference in 24h-UCa levels between PHPT patients with and without kidney stones across all subgroups (**Table 2**). However, a significant difference in 24h-UCa levels was observed between genders, with men having higher 24 h-UCa levels than women (p <0.05), but the gender difference became insignificant when considering the presence of kidney stones. Additionally, 24h-UCa levels were significantly higher in white people compared to black people (**Table 2**), although this racial difference was only marginally significant in patients with kidney stones (p = 0.075).

**Table 2:** 24-hour urinary calcium levels by stone presence and demographic group.

Variable	24h-UCa (mg/day)			p^1^
	Overall	No Stone	Stone	
Female	240 ± 159 (240)	239 ± 159 (195)	247 ± 162 (45)	0.732
Male	297 ± 201 (66)	279 ± 198 (45)	334 ± 335 (21)	0.229
p^2^	0.042	0.179	0.115	
Black people	190 ± 139 (104)	187 ± 145 (88)	203 ± 102 (16)	0.309
White people	284 ± 176 (202)	281 ± 170 (152)	298 ± 195 (50)	0.615
p^3^	<0.001	<0.001	0.075	

The data of 24h-UCa expressed as mean ± SD (sample size).

p1: Compare 24h-UCa between groups by stone presence.

p2: Compare 24h-UCa between groups by gender.

p3: Compare 24h-UCa between groups by race.

**Table 3** shows that 42% of the overall PHPT patients had hypercalciuria. The level classification of 24 h-UCa for all patients presents the prevalence of hypercalciuria (hUCa) across genders, races, and kidney stones. There was no significant difference in hypercalciuria prevalence between men and women, or among individuals with and without kidney stones. However, a significant racial difference was found (p < 0.001) with hypercalciuria being more prevalent in white people compared to black people (**Table 3**).

**Table 3:** The prevalence of hypercalciuria across genders, races and stones.

Variable	24h-UCa Level Classification		p
	nUCa	hUCa	
	177 (58%)	129 (42%)	
Gender			
Men	37 (21%)	29 (22%)	0.741
Women	140 (79%)	100 (78%)	
Race			
Black people	76 (43%)	28 (21%)	<0.001
White people	101 (57%)	101 (78%)	
Stones			
Yes	33 (50%)	33 (50%)	0.146
No	144 (60%)	96 (40%)	

The data expressed as case-number (percent).

nUCa, normocalciuria; hUCa, hypercalciuria.

**Table 4** presents the results of multivariate logistic regression analysis. This model examined whether gender, race, age, and 24h-UCa level independently predicted kidney stone formation in PHPT patients, adjusting for all variables simultaneously. The results demonstrated that male gender (OR = 2.28, 95% CI 1.19–4.37, p = 0.013) and age > 65 years (OR = 1.89, 95% CI 1.05–3.42, p = 0.034) were identified as independent predictors of kidney stone formation in PHPT patients. Race and 24h-UCa level were not significantly associated with stone risk after adjustment (p > 0.05).

**Table 4:** Predictors of kidney stones in PHPT patients.

Variable	Assignment	Coefficient	Std. Error	Odds ratio	95% CI	p
Gender	Female=0, Male=1	0.824	0.333	2.28	1.19-4.37	0.013
Race	Black=0, White=1	-0.447	0.331	0.64	0.33-1.22	0.177
Age	¾65 = 0, >65 = 1	0.639	0.302	1.89	1.05-3.42	0.034
24h-UCa	Low=0, High=1	0.213	0.294	1.24	0.69-2.20	0.469

The data are analyzed using Multivariate Logistic Regression.

**Table 5** presents the results of a multiple regression analysis examining the relationship between various demographic and serum biochemical factors and 24-hour urinary calcium (24h-UCa) levels. The analysis identified several significant predictors of high 24h-UCa, including race (p < 0.001), gender (p = 0.004), eGFR (p < 0.001), and serum calcium levels (p = 0.002). Age, serum creatinine, PTH, 25(OH)D, and stone history were not significant after adjustment. When applying the Benjamini–Hochberg false discovery rate (q = 0.05), these four associations remained significant (their p-values were ≤ the BH critical values 0.0056, 0.011, 0.017, and 0.022, respectively); all other predictors were not significant after FDR correction. The R^2^ = 0.249 and R^2^-Adjisted = 0.226. The variance inflation factor (VIF) was all <2.5, indicating that multicollinearity was not an issue.

**Table 5:** Demographic and serum biochemical factors associated with urine calcium levels.

24h-UCa	Coefficient	Std. Error	t	P	VIF
Age	-1.48	0.983	-1.51	0.132	1.51
Gender	64.1	21.9	2.93	0.004	1.07
Race	101	19.1	5.29	<0.001	1.08
Stones	-8.36	21.9	-0.382	0.703	1.06
Calcium	51.4	16.3	3.16	0.002	1.27
Creatinine	-3.08	2.45	-1.26	0.210	1.02
GFR	2.30	0.535	4.31	<0.001	1.51
PTH	0.195	0.170	1.15	0.250	1.31
25(OH)D	-0.228	0.688	-0.332	0.740	1.11

The data are analyzed using Multiple Regression Analysis R2 = 0.249; R2-Adjusted = 0.226.

## 4 Discussion

The prevalence of kidney stones in our large, well-characterized, and surgically confirmed patients with PHPT was 22%, which was similar to or significantly lower than the rates reported by other studies (5, 6, 18, 19). However, the prevalence of kidney stones in our PHPT individuals is still notably higher than the 10% in the general American population (20), emphasizing the potential role of PHPT in predisposing individuals to kidney stone formation. Male patients with PHPT were more likely to develop kidney stones (32%) than female patients (19%), a pattern consistent with the general population but more pronounced (20, 21). Racial differences were also observed, with the expected higher prevalence among white people (37%) than black people (24%). Our study indicated that patients with PHPT who had kidney stones were significantly older than those without stones. These findings suggest that demographic factors, such as age, gender, and race, are more important for kidney stone risk in PHPT, although the underlying mechanisms remain unclear. In contrast, none of the serum biochemical variables showed significant differences between patients with and without kidney stones.

Published data on the utility of 24 h-UCa as a predictor of kidney stone formation in PHPT have been inconsistent (11–14, 22). Variations in patient inclusion criteria, methods for diagnosing kidney stones and techniques for measuring 24 h-UCa, as well as differing dietary and lifestyle factors, can all lead to conflicting results that complicate the overall understanding of this issue (5, 12, 14, 23–30). In our study, we only included patients with hypercalcemic PHPT with serum Ca levels higher than the reference range (10.2 mg/dL) (26) and patients of different genders and races. Across all subgroups, no significant difference in 24h-UCa levels was observed between the patients with and without kidney stones. This lack of association persisted even after adjusting for factors such as age, gender, and race in the multivariate logistic regression analysis, suggesting that 24h-UCa is not a reliable standalone predictor of kidney stone formation as previously reported (11, 31). However, the multivariate logistic regression model demonstrated that gender and age were independently significant predictors of changes in 24h-UCa levels.

Our study indicated that demographic factors were dominant predictors of 24h-UCa levels in PHPT patients as in the general population. Men exhibited higher urinary calcium excretion than women (10, 32), although this difference was insignificant when considering the presence or absence of kidney stones. Similarly, white individuals showed significantly higher 24h-UCa levels and a greater prevalence of hypercalciuria than black individuals (33). These findings highlight the influence of genetic, hormonal, and environmental factors on calcium metabolism and kidney stone risk (34, 35). Notably, racial differences in hypercalciuria and kidney stone prevalence (22, 36) underscore the need for demographic-specific approaches to PHPT management.

The occurrence of hypercalciuria in patients with PHPT is somewhat paradoxical, since the primary renal effect of PTH is to increase renal tubular reabsorption Ca (TRCa). Thus, for hypercalciuria to occur in PHPT, the PTH-dependent increased TRCa must be overcome by the increased filtered load, which usually does not occur until serum Ca level exceeds 12 mg/dl (12, 37, 38). Alternatively, some patients with PHPT may have unrecognized co-existent renal tubular leak hypercalciuria, which explains the variable response of nephrolithiasis and hypercalciuria to curative PTX (38, 39). In addition, there is good evidence to suggest that patients with PHPT have an increased intestinal absorption of calcium due to PTH-mediated increased production of 1,25-dihydroxyvitamin D (40, 41). However, the relative contributions of the three mechanisms (i.e., increased filtered load, renal tubular leak, and increased intestinal absorption of Ca) to hypercalciuria in patients with PHPT are not well studied and require further research (38, 39, 42–44).

This study has several limitations, most of which are related to its retrospective design. First, our cohort was limited to patients with surgically confirmed PHPT, which may limit the generalizability of the findings to patients with mild or normocalcemic disease who are not surgical candidates. Second, kidney stone status was determined through medical history, record review, and available imaging, which may have missed silent or remote stones and could not adequately distinguish between past and recent stone events. Such non-differential misclassification would likely bias results toward a null hypothesis and may explain weak associations. Third, we relied on a single 24-hour urine collection closest to surgery, an approach that is vulnerable to collection errors and day-to-day variability, introducing random noise that may reduce statistical power. Fourth, we lacked detailed dietary and urinary data, including calcium intake, and urine oxalate and citrate measurements. Although 24-hour urinary sodium was measured, extensive missing data precluded its inclusion in the models, limiting our ability to adjust for the known effect of urine sodium on calcinuria. Finally, race was abstracted from the medical record and categorized as “Black” or “White,” a social rather than biological classification that cannot separate genetic, hormonal, cultural, or socio-environmental influences.

Collectively, these limitations likely increased random variability, which would tend to diminish rather than exaggerate observed associations. Therefore, our null findings should be considered conservative. Future prospective studies with standardized dietary assessments, repeated urine collections, genetic ancestry data, and social determinant measures are needed to more precisely define predictors of urinary calcium excretion and stone risk across the full spectrum of PHPT.

## 5 Conclusion

Our study shows that 24h-UCa is not a reliable predictor of kidney stone formation in patients with PHPT. No significant differences in 24h-UCa levels were found between stone-formers and non-stone-formers. In contrast, demographic factors—especially gender and race—were strong indicators of urinary calcium excretion, with men and white individuals exhibiting higher levels and a greater prevalence of hypercalciuria. The positive relationships of 24h-UCa with serum calcium and eGFR highlight the role of systemic calcium metabolism but also present the limitations of using 24h-UCa alone as a diagnostic tool. Future research should investigate additional urinary risk factors that may contribute to stone formation in PHPT. Overall, the routine use of 24h-UCa to guide parathyroidectomy decisions should be reconsidered.

## Data Availability

The original contributions presented in the study are included in the article/supplementary material. Further inquiries can be directed to the corresponding author.
